# Comprehensive Analysis of Mutations in the Hepatitis Delta Virus Genome Based on Full-Length Sequencing in Individuals Infected with Genotype 3 in Brazil

**DOI:** 10.1007/s00705-026-06697-z

**Published:** 2026-07-30

**Authors:** Tárcio Peixoto Roca, Jackson Alves da Silva Queiroz, Ana Maisa Passos-Silva, Mayara Torquato Lima da Silva, Eugênia de Castro-Silva, Lourdes Maria Pinheiro Borzacov, Juan Miguel Villalobos-Salcedo, Deusilene Vieira, Livia Melo Villar

**Affiliations:** 1 Laboratório de Hepatites Virais, Instituto Oswaldo Cruz, FIOCRUZ, Rio de Janeiro, RJ Brazil; 2 bLaboratório de Virologia Molecular, Fundação Oswaldo Cruz Rondônia, FIOCRUZ-RO, Porto Velho, RO Brazil; 3 Programa de Pós-Graduação em Biologia Experimental, Universidade Federal de Rondônia - UNIR, Porto Velho, RO Brazil; 4 Laboratório de Biotecnologia e Bioengenharia Estrutural, Instituto de Biofísica Carlos Chagas Filho, UFRJ, Rio de Janeiro, Brazil; 5 Centro de Pesquisa em Medicina Tropical - CEPEM, Ambulatório de Hepatites Virais, Porto Velho, RO Brazil

## Abstract

HDV has a high genetic diversity among isolates and a high capacity to accumulate mutations over time. The mechanisms involved in the clinical progression of HDV are not fully elucidated. Few studies relate mutations to clinical implications, which makes this variable extremely relevant for research into HDV-3, a genotype associated with worse clinical progression. The aim of this study was to analyze mutations in complete genome sequences of HDV-3 isolated in the Brazilian Western Amazon. During 2010 to 2023, 35 hepatitis Delta patients with mild or advanced fibrosis gave serum samples that were submitted to conventional PCR and sequencing. Synonymous and non-synonymous mutations were analyzed along the delta antigen sequence based on the reference strain NC_076103.1. N-terminal and C-terminal regions of Large HDAg, molecular modeling and docking analyses were performed. 114 non-synonymous substitutions were identified in 76 positions along the HDAg-L. To synonymous mutations, 46 substitutions were found in 44 nucleotide sites. S/L8P/Q/M was the most frequent mutation among the advanced fibrosis group. The D46E and A70T mutation was more observed among patients with increased AST. These associations showed statistically significant p-values. Mutations in the N-terminal region of Large HDAg led to minor conformational changes. The C-terminal region indicates moderate structural differences caused by the mutations. The binding conformation of Large HDAg with Mutant2C exhibiting the most stable interaction, as suggested by its docking score. This study shows some evidence of the influence of HDAg mutations in relation to clinical significance, providing insights for further, longitudinal research.

**Supplementary Information:**

The online version contains supplementary material available at 10.1007/s00705-026-06697-z.

## Background

Hepatitis delta virus (HDV) is a hepatotropic virus responsible for the most severe form of viral hepatitis in humans [1]. As HDV is considered to be a defective virus, infection is only possible when it coexists with the hepatitis B virus (HBV), which needs its surface proteins (HBsAg) to penetrate hepatocytes [2, 3].

HDV measures about 36 nm in diameter with a circular ssRNA (-) genome of approximately 1.7 kb [4] responsible for encoding only one protein (HDAg), which can be presented in two isoforms: S-HDAg and L-HDAg [5, 6]. Thus, a total of eight HDV genotypes have been isolated to date and are identified in numerical order from 1 to 8 (HDV-1/HDV-8) [7–10].

HDV-3 has been identified as the causative agent of outbreaks of severe and frequently fulminant hepatitis in northeastern South America [11]. Epidemiological studies indicate that this genotype is highly prevalent in the Brazilian Amazon, where it has been consistently associated with a more aggressive clinical course and increased disease severity [12–14]. It was analyzed that HDV-3 has a high genetic diversity among the isolates, with a substitution rate of 8.2×10^4^ substitutions/site/year [15], demonstrating that this genotype has a high capacity to accumulate mutations over time and high intragenotypic variability [16].

The mechanisms involved in the clinical progression of HDV are not fully elucidated due to the involvement of multiple factors [16]. Few studies relate mutations to clinical implications, which makes this variable extremely relevant for research into HDV-3, a genotype strongly associated with clinical progression to disease severity. Thus, the aim of this study was to analyze mutations in complete genome sequences of HDV-3 isolated in the Brazilian Western Amazon.

## Methods

*Ethical Aspects*

The research procedures were carried out in accordance with the ethical principles stipulated by the World Medical Assembly of 1975 and by the Brazilian Ministry of Health and follow the norms established in Resolution No. 466 of December 12, 2012. This research was approved by the research ethics committee of Research Center in Tropical Medicine of Rondônia - CEPEM/RO (CAAE No. 17616019.1.0000.0011) and informed consent was obtained from all participants.

### Study Population

The samples used in this study were acquired from a previous study conducted by Roca et al. (2023), comprising 35 serum samples obtained from individuals positive for hepatitis Delta (anti-HDV). The sampling period was from 2010 to 2023, from the Viral Hepatitis Outpatient Clinic of the CEPEM, which cares for patients from Rondônia and Amazonas States (North Region of Brazil). Inclusion criteria were: a) diagnosis of hepatitis D superinfection (positive for total Anti-HBc and HBsAg for over six months, anti-HDV positive); b) detectable HDV-RNA; c) both genders; and d) age between 18 and 70 years old. Exclusion criteria included immunosuppression, pregnancy, co-infection with HCV and/or HIV, and no informed consent.

### Clinical Aspects

All demographic, laboratory and clinical records were obtained from medical records. For clinical evaluation, study participants were classified according to degrees of fibrosis using the METAVIR score: (i) Individuals without fibrosis or mild fibrosis (F0/F1) (ii) Individuals with advanced fibrosis (F3/F4), as determined by (1) a biopsy (2) elastography according to the degree of fibrosis.

### Acid Nucleic Extraction and RNA-HDV Detection

All the samples were subjected to the genetic material extraction procedure (RNA-HDV) using the EXTRACTA KIT FAST-Viral DNA/RNA Kit (MVXA-P016FAST) in the EXTRACTA 32 automated DNA and RNA extractor (LOCCUS, São Paulo, Brazil). The procedure was carried out according to the manufacturer's instructions, and the final volume of purified RNA was 50 µL. For detection of HDV-RNA, extractions were subjected to the RT-qPCR protocol developed by Queiroz et al [17].

### Reverse Transcription

The purified RNA positive in RT-qPCR was subjected to reverse transcription using the SuperScript™ III Reverse Transcriptase enzyme (Thermo Fisher Scientific®, Waltham, MA, USA) and random primers to synthesize complementary DNA (cDNA) according to the manufacturer's instructions.

### Conventional PCR and Sanger Sequencing

Full coverage of the HDV genome was obtained by amplifying two fragments (966p and 126pb) from a conventional PCR previously described by Angelice el al., 2024 [18] and adapted by Roca et al., 2024 [15]. The first fragment, approximately 966 bp in length, was amplified using the sense primer 320 s (5′ CCAGAGGACCCCTTCAGCGAAC 3′) and the antisense primer 1267as (5′ GAAGGAAGGCCCTGGAGAACAAGA 3′). The PCR protocol consisted of an initial denaturation at 98 °C for 30 s, followed by 40 cycles of 98 °C for 15 s, 60 °C for 30 s, and 72 °C for 45 s, and a final extension step of 5 min at 72 °C. The second fragment, measuring roughly 1216 bp, was generated with primers 900 s (5′ CATGCCGACCCGAAGAGGAAAG 3′) and 503as (5′ CCCCGGGATAAGCCTCACTCG 3′). Amplification was performed with an initial denaturation at 98 °C for 30 s, followed by 40 cycles at 98 °C for 15 s, 58 °C for 30 s, and 72 °C for 45 s, and concluded with a 5-min final extension at 72 °C.

The PCR products were purified using the ExoSAP-IT PCR Product Cleanup Kit (Applied Biosystems™, Foster City, CA, USA). Positive samples were sequenced by the automated Sanger method using the BigDye™ Terminator v1.1 Cycle Sequencing Kit (Applied Biosystems™, CA, USA) according to the manufacturer's instructions. The reaction product was purified using the BigDyeXTerminator™ Purification Kit (Applied Biosystems™, CA, USA). The run was carried out by the FIOCRUZ Technology Platforms Network RPT01N-FIOCRUZ/RO using an automated Sanger Seqstudio sequencer (Applied Biosystems, Waltham, MA, USA).

### Mutation Analysis

Editing and assessing the quality of the electropherograms to obtain the consensus sequence was carried out using MEGA v.11.0 software [19]. The nucleotide and proteins sequences were aligned for mutation analysis using the MUSCLE algorithm [20] with a sequence of Large Delta antigen (HDAg-L) of the reference strain NC_076103.1 to provide the origin and location of the amino acids, as well as analyzing possible amino acid changes in the sequenced samples.

### Structural and Molecular Docking Analysis of Large HDAg Mutations

To evaluate the impact of mutations in the N-terminal (residues 12–60) and C-terminal (residues 195–214) regions of Large HDAg, molecular modeling and docking analyses were performed (table 1). Three mutated peptides were selected for the N-terminal region (R13K, A/I16V/T, K27R, I/T37A/C/S), modeled via SwissModel using PDB: 1A92 as a reference. Structural analyses in PyMOL assessed root-mean-square deviation (RMSD), solvent-accessible surface area (SASA), and intermolecular interactions) [21]. For the C-terminal region, three peptides with mutations (F193Y, F198L, Y205H, V208A) were modeled using I-TASSER [22]. Mutants were aligned in PyMOL, and molecular docking with HBsAg (PDB: 9IYX) was performed via HADDOCK v.2.4 [23]. Binding affinities with the HBsAg pre-S1 region (peptide 17HQLDPAFG24, PDB: 1KC5) were analyzed using docking scores. The sequences of the mutated peptides for N-terminal and C-terminal regions are as follows:

**Table 1.** Mutated Peptides in the N-terminal and C-terminal Regions

Peptide	Mutation	Sequence (12–60)
MutantN1	R13K, A/I16T, K27R, I/T37C	GKEDTLEQWVSGRKRLEELERDLRCLKKKIKKLEEENPWLGNIKGIIGKY
MutantN2	R13K, A/I16V, K27R, I/T37S	GKEDVLEQWVSGRKRLEELERDLRSLKKKIKKLEEENPWLGNIKGIIGKY
MutantN3	R13K, A/I16V, K27R, I/T37A	GKEDVLEQWVSGRKRLEELERDLRALKKKIKKLEEENPWLGNIKGIIGKY
Peptide	Mutation	Sequence (195–214)
MutantC1	F193Y, F198L, Y205H, V208A	YPWYGLTPPPPGHYWAPGCTQQX
MutantC2	F193Y, F198L, Y205H	YPXYGLTPPPPGHYWVPGCTQQX
MutantC3	F198L, Y205H, V208A	FPXYGLTPPPPGHYWAPGCTQQX

### Statistical Analysis

Frequencies and measures of central tendency and dispersion were used for descriptive statistics. To investigate the associations between the identified mutations and clinical/laboratory outcomes, we used multivariate logistic regression models, adjusting for relevant covariates (gender and age) and potential confounding factors. All tests were carried out considering a value of p < 0.05 as significant. The analyses and graphs were carried out using the JASP v0.95.4 and R v4.4.2 software [24].

## Results

A total of 35 patients with HDV infection were included in the study and classified as HDV-3. A total of 54.3% (19/35) of the individuals had advanced fibrosis and, of these, 8.6% (3/35) were classified as having cirrhosis. None were diagnosed with hepatocellular carcinoma. Table 2 shows the results of the demographic, laboratory, clinical and virological characterization of the study population.

**Table 2.** Description of the study population

Variable	Value
Demographic data	
Male/Female	21/14
Age (years, Mean ± SD)	38 ± 12
Laboratory data	
HBeAg positive (n, %)	5 (14.3%)
Anti-Hbe positive (n, %)	32 (91.4%)
ALT (U/L, Mean ± DP)	46 ± 67.7
AST (U/L, Mean ± DP)	50.5 ± 52.5
RNA-HDV Viral Load (Log 10 copies/ml, Mean ± DP)	5.6 ± 1.1
Clinical data	
Mild fibrosis (n, %)	16 (45.7%)
Advanced fibrosis (n, %)	19 (54.3%)
Treatment (n, %)	19 (54.3%)

Abbreviations: ALT - alanine aminotransferase; AST - aspartate aminotransferase

**Figure 1.**
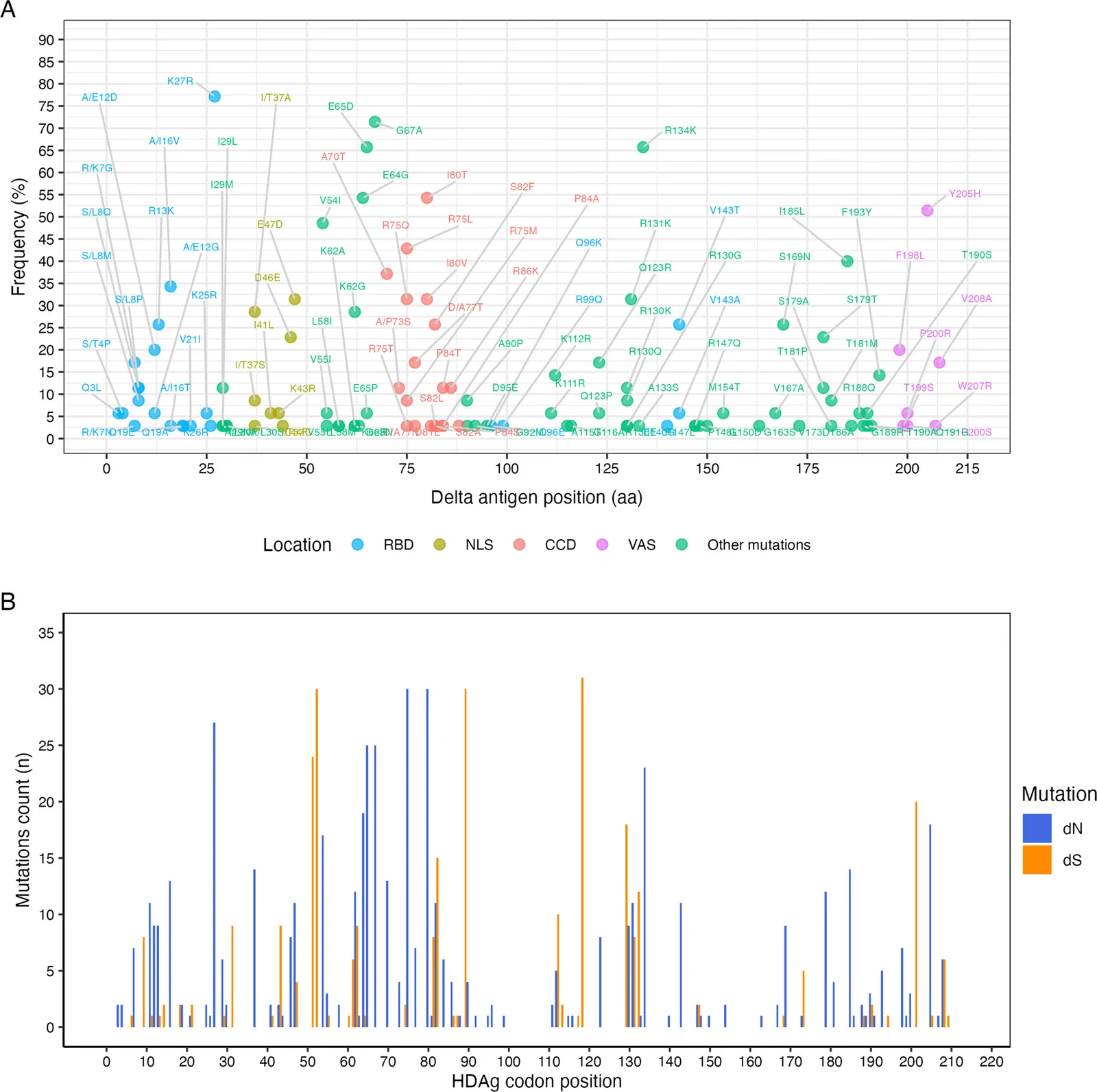
(**A**) Dot plot showing the frequency of mutations identified in the 33 samples analyzed. Mutation analysis was carried out based on comparison with the reference sequence NC_076103.1 for HDV-3. (**B**) Bar plot showing the number of synonymous and non-synonymous mutations identified in the study at each HDAg codon position. Abbreviations: RBD - RNA-binding domain; NLS - nuclear localization signal; CCD - coiled coil domain; VAS - virus assembly signal; dN - Non-synonymous mutations; dS – Synonymous mutations.

In the analysis of HDV-3 mutations, 114 amino acid substitutions were identified, distributed in 76 different positions within the main regions of the delta antigen (figure 1A). Among the mutations located in important HDAg domains, the substitution of the amino acid lysine (K) for arginine (R) at position 27 (K27R) was the most frequent, present in 77.1% (27/35) of the samples analyzed. Next, the substitution of isoleucine (I) for threonine (T) at position 80 (I80T) was observed in 48.6% (17/35) of the samples. The mutation of tyrosine (Y) to histidine (H) at position 205 (Y205H) occurred in 45.7% (16/35) of the samples, while the substitution of arginine (R) for leucine (L) at position 75 (R75L) was detected in 37.1% (13/35) of the samples.

In relation to synonymous mutations, 46 substitutions were found in 44 nucleotide positions of the HDAg sequence that did not culminate in an amino acid change. Figure 1B shows the number of synonymous and non-synonymous mutations identified along the codons of the delta antigen.

Patients with mild and advanced fibrosis were included in comparison with the mutations in the main HDAg regions. This information has been provided in Table 3 in comparison with the mutations that were identified in each patient.

**Table 3.** Characteristics of samples with mutations in relevant HDAg regions

Sample ID	Sex	Treatment	Fibrosis	HDAg mutations	GenBank ID
HDVAR-1	Female	ETV	Advanced	K27R, V54I, V55L, R75Q, P84T, A90P, Q123R, S169N	PQ220458
HDVAR-2	Female	ETV	Advanced	V54I, E65D, A/P73S, R75Q, I80V, P84A, R86K, A90P, G92M, Q123R, R130Q, E140G, S169N, I185L	PQ220459
HDVAR-3	Female	No	Mild	K25R, K27R, I29N, G67A, R75Q, D/A77T, I80V, P84S, R86K, Q96E, V143A, R147L, 169 N, T181M, F198L	PQ220460
HDVAR-4	Male	PEG-IFN-α + TDF	Advanced	S/T4P, R/K7N, S/L8P, A/E12D, A/I16V, K27R, I41L, D63N, R75Q, D/A77T, I80V, S82F, D95E, A115T, G116A, A133S, V143T, F198L, Y205H	PQ220461
HDVAR-5	Male	No	Mild	R13K, A/I16V, K27R, I29M, V54I, E64G, E65D, G67A, R75L, I80T, R131K, S179T, I185L, F193Y	PQ220462
HDVAR-6	Female	PEG-IFN-α + ETV	Advanced	K27R, K62G, E64G, E65D, G67A, A70T, R75L, I80T, S82F, K112R, Y205H	PQ220463
HDVAR-7	Female	No	Mild	Q3L, R/K7G, A/E12D, V21I, K27R, A/L30S, I/T37S, E47D, K62R, E65P, G67A, R86K, R134K, V143A, Q191G, F198L, P200R	PQ220464
HDVAR-8	Male	ETV	Advanced	S/L8Q, A/E12D, A/I16V, I41L, R75Q, D/A77T, I80V, K111R, M154T, V167A, S169N, T181M, I185L, Y205H, V208A	PQ220465
HDVAR-9	Male	PEG-IFN-α + ETV	Advanced	R/K7G, S/L8Q, A/E12G, I/T37A, S82F, A90P, Q123R, R130K, G150D, S169N, I185L, T190A	PQ220466
HDVAR-10	Male	PEG-IFN-α + ETV	Advanced	S/L8Q, A/E12D, K27R, V54I, L58M, A/P73S, R75Q, D/A77T, I80V, S82F, P84T, R86K, A90L, Q123R, R130E, V143T, R147Q, P148L	PQ220467
HDVAR-11	Male	No	Mild	S/L8Q, K27R, I29L, I/T37S,L58I, R75L, D/A77T, I80V, P84T, R130Q, F193Y, T199S, P200R, Y205H, V208A	PQ220468
HDVAR-12	Female	PEG-IFN-α + ETV	Advanced	S/T4P, A/E12G, Q19E, K27R, I/T37C, K62A, E65P, G67A, I80V, Q123R, R130Q, V143T, F198L, V208A	PQ220469
HDVAR-13	Male	ETV	Advanced	S/L8Q, R13K, Q19A, K27R, I29M, I/T37S, V54I, V55I, E65D, G67A, A/P73S, R75T, D/A77N, Q123R, R131K, V143T, W207R	PQ220470
HDVAR-14	Female	ETV	Advanced	S/L8M, A/E12D, R13K, K25R, K27R, I29M, A/L30F, E47D, V54I, V55I, G67A, R75Q, I80T, S82A, S/P88K, Q96K, R99Q, Q123P, V143T, G163S, I185L, G189R, T190S, F198L, V208A	PQ220471
HDVAR-15	Male	No	Mild	R/K7G, A/I16V, K27R, E47D, V54I, E64G, E65D, A70T, R75L, I80T, D81E, K111R, R131K, V167A, S169N, I185L	PQ220472
HDVAR-16	Male	PEG-IFN-α	Advanced	S/L8P, A/E12D, L44F, D46E, E65D, A/P73S, R75Q, I80V, V143T, T181P, I185L, D186A, R188Q, T190S, F198L	PQ220473
HDVAR-17	Male	No	Mild	S/L8M, A/I16V, E47D, V54I, E65D, R75Q, I80V, V143T, S169N, S179A, I185L, V208A	PQ220474
HDVAR-18	Male	ETV	Advanced	S/L8M, A/I16V, K27R, E47D, V54I, E65D, R75Q, I80V, V143T, S169N, S179A, I185L, V208A	PQ220475
HDVAR-19	Male	PEG-IFN-α + ETV	Mild	A/E12D, A/I16T, I/T37A, D46E, E47D, V54I, G67A, R75Q, D/A77T, I80V, P84T, V143T, M154T, S169N, V173I, I181M, I185L, F198L, Y205H	PQ220476
HDVAR-20	Male	PEG-IFN-α + ETV	Advanced	A/I16V, I/T37A, E47D, K62G, E64G, E65D, G67A, A70T, R75L, I80T, K112R, Y205H	PQ220477
HDVAR-21	Male	No	Mild	R13K, K26R, K27R, I/T37A, V54I, E64G, G67A, R75L, I80T, Q123P, R130G, I185L, R188Q	PQ220478
HDVAR-22	Female	No	Mild	R/K7G, R13K, A/I16V, K27R, I/T37A, D46E, V54I, E64G, E65D, G67A, R75M, I80T, R130K, R131K, S179T, I185L, F193Y, Y205H	PQ220479
HDVAR-23	Male	ETV	Advanced	R/K7G, R13K, A/I16V, K27R, I/T37A, D46E, V54I, E64G, E65D, G67A, R75M, I80T, R130K, R131K, S179T, I185L, F193Y, Y205H	PQ220480
HDVAR-24	Male	No	Mild	R/K7G, R13K, A/I16V, K27R, I/T37A, D46E, V54I, E64G, E65D, G67A, R75M, I80T, R130K, R131K, S179T, I185L, F193Y, Y205H	PQ220481
HDVAR-25	Male	No	Mild	R13K, K27R, E64G, E65D, G67A, A70T, R75L, S82F, R131K, Y205H	PQ220482
HDVAR-26	Male	No	Mild	R13K, K27R, D46E, E64G, E65D, G67A, A70T, R75L, I80T, S82F, R131K	PQ220483
HDVAR-27	Female	PEG-IFN-α	Advanced	K27R, E47D, E64G, E65D, G67A, R75L, I80T, R131K	PQ220484
HDVAR-28	Male	No	Mild	K27R, I/T37A, E47D, K62G, E64G, E65D, G67A, A70T, R75L, I80T, S82F, K112R, Y205H	PQ220485
HDVAR-29	Male	PEG-IFN-α + ETV	Advanced	A/I16V, K27R, I/T37A, E47D, K62G, E64G, E65D, G67A, A70T, R75L, I80T, S82F, K112R, Y205H	PQ220486
HDVAR-30	Female	PEG-IFN-α + ETV	Advanced	I/T37A, K62G, E64G, E65D, G67A, A70T, R75L, I80T, S82F, K112R, Y205H	PQ220487
HDVAR-31	Female	No	Advanced	Q3L, K27R, V54I, K62G, E64G, E65D, G67A, A70T, R75L, I80T, S82L, R131K, S179A, P200S, Y205H	PQ220488
HDVAR-32	Female	PEG-IFN-α	Advanced	K27R, V54I, K62G, E64G, E65D, G67A, A70T, R75L, I80T, K112R, Y205H	PQ220489
HDVAR-33	Male	No	Mild	S/L8P, K27R, E47D, V54I, E64G, E65D, G67A, A70T, R75L, I80T, R131K, S179A, Y205H	PQ220490
HDVAR-34	Female	No	Mild	K27R, D46E, K62G, E64G, E65D, G67A, A70T, I80T, Y205H	PQ220491
HDVAR-35	Female	No	Mild	A/I16V, K27R, D46E, K62G, E64G, E65D, A70T, I80T, Y205H	PQ220492

Abbreviations: ETV – Entecavir; PEG-IFN-α - Pegylated interferon-α; TDF - Tenofovir disoproxil fumarate.

**Figure 2.**
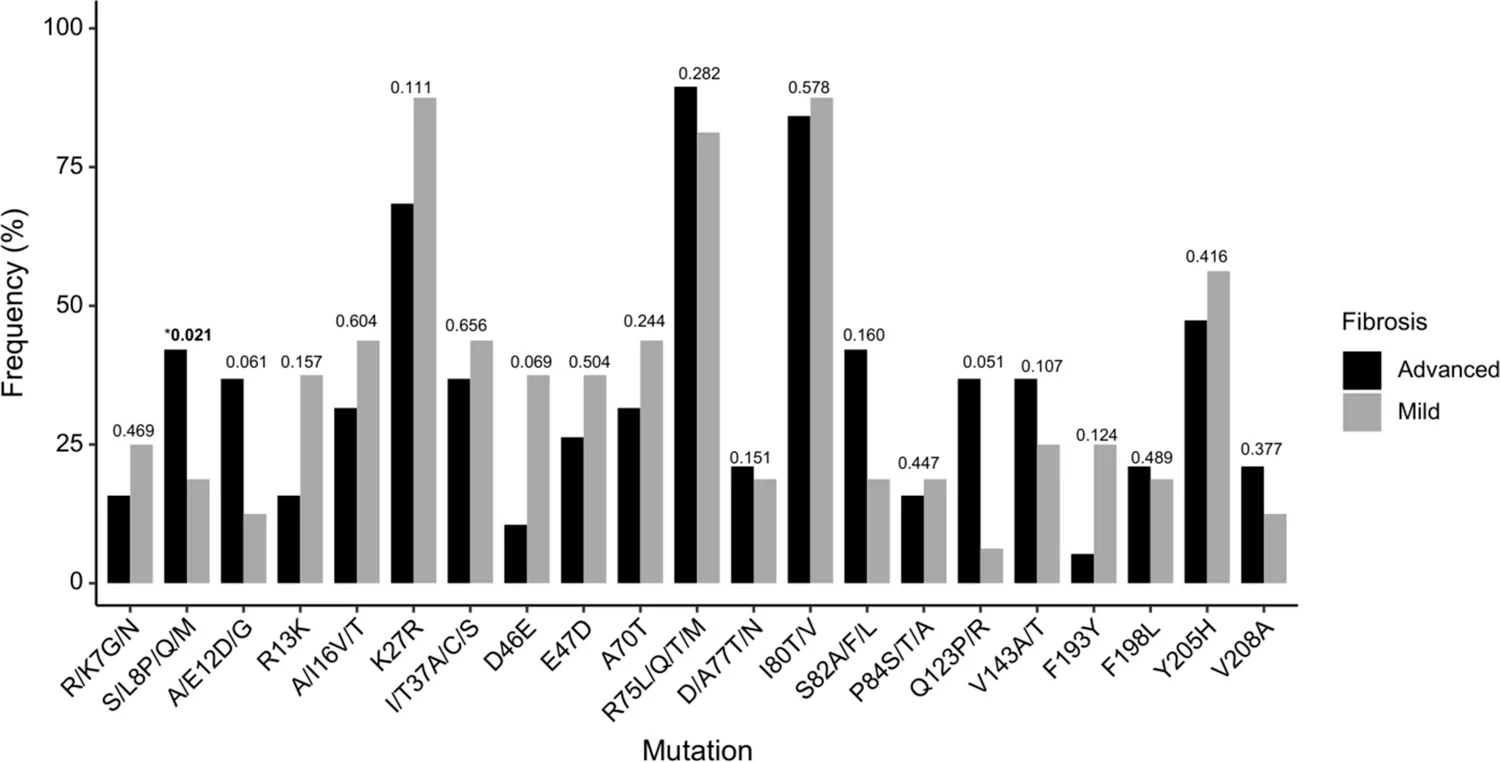
Bar plot showing the frequency of individuals in each fibrosis group in relation to 21 most frequent positions of HDAg-L. The numbers of patients in each fibrosis group are found above the bars. Significant p-values (p < 0.05) are highlighted in bold with an asterisk. The p-values shown are adjusted for the covariates age and sex according to the logistic regression model

Mutations in 21 of the most frequent HDAg-L positions were analyzed (figure 2). Among the mutations identified, the substitution S/L8P/Q/M was found to be most frequent in the group of patients with statistically significant advanced fibrosis (p=0.021).

**Figure 3.**
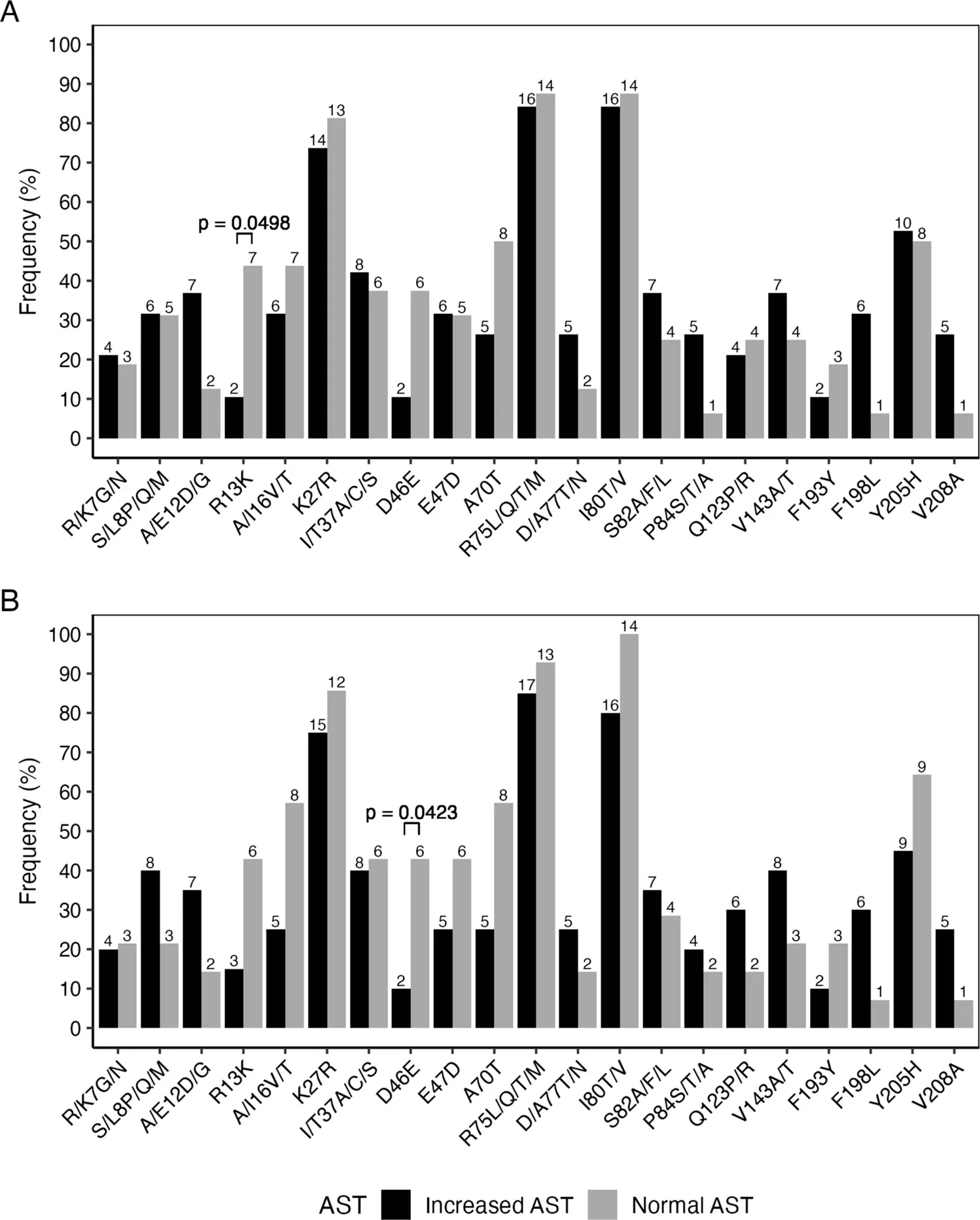
Comparison of the main mutations found in relation to ALT (A) and AST (B) levels group. The numbers of patients in each ALT and AST group are found above the bars. AST and ALT reference values: 10–40 U/L. Significant p-values (p < 0.05) are highlighted in bold with an asterisk. The p-values shown are adjusted for the covariates age and sex according to the logistic regression model.

The proportion of the most frequent mutations was compared in relation to the groups of patients who showed elevated ALT and AST. The D46E and A70T mutations was frequent among patients with normal AST (figure 3). These observations had a significant p-value.

**Figure 4.**
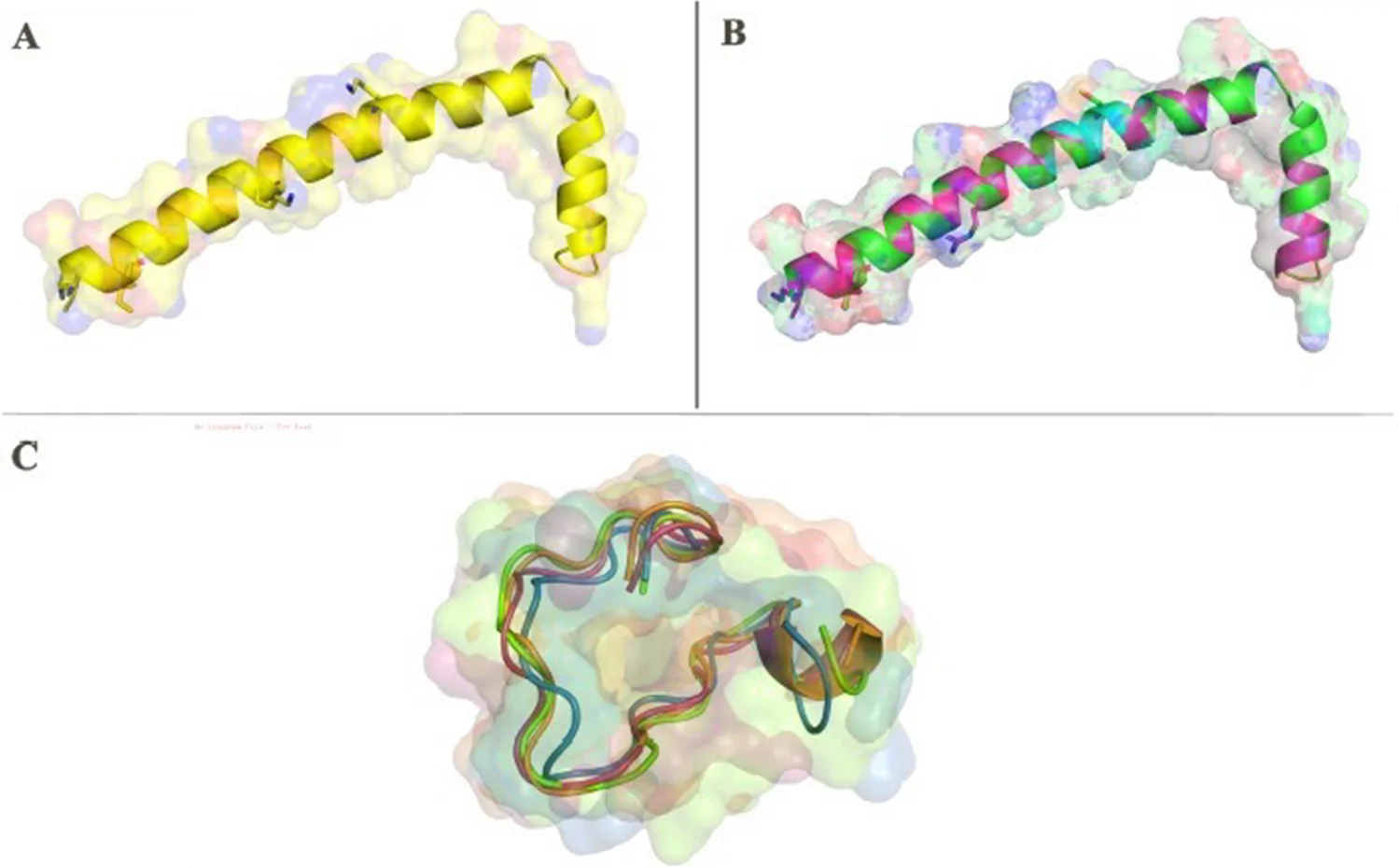
Structural analysis of Large HDAg peptides. A) Reference structure of the N-terminal region (residues 12–60) (PDB: 1A92). (B) Structural alignment of mutated N-terminal peptides, with an average RMSD of 0.094 Å. The solvent-accessible surface area (SASA) of the reference peptide is 5500.78 Å^2^, while the mutated peptides exhibit an average SASA of 5554.66 ± 6.8 Å^2^. (C) Structural overlay of the C-terminal region, showing the reference structure (pink) and mutants (Mutant1C in green, Mutant2C in blue, and Mutant3C in orange). Surface representation generated in PyMOL highlights structural volume differences, with an RMSD of 2.471 Å between the structures.

The N-terminal region of Large HDAg plays a crucial role in dimerization and RNA binding, directly influencing HDV replication (4, 5). Structural analysis revealed that the introduced mutations led to minor conformational changes. When compared to the reference peptide, the RMSD values were 0.095 Å for mutantN1, 0.094 Å for mutantN2, and 0.094 Å for mutantN3, indicating minimal deviation from the original structure. The solvent-accessible surface area (SASA) of the reference peptide was 5500.755 Å^2^, whereas the mutated peptides exhibited SASA values of 5561.056 Å^2^ (mutantN1), 5556.552 Å^2^ (mutantN2), and 5546.001 Å^2^ (mutantN3), suggesting slight alterations in the structural conformation that may influence protein interactions (Figure 4, A and B).

Conversely, the C-terminal region, which differentiates Large HDAg from Small HDAg, is crucial for interaction with HBsAg and viral particle formation (6). Structural analysis revealed moderate conformational differences among the mutated peptides when compared with the reference structure. The RMSD values were 2.396 Å for mutantC1, 3.020 Å for mutantC2, and 1.997 Å for mutantC3 relative to the reference peptide. These deviations indicate that the C-terminal mutations produce noticeable but localized structural alterations. Figure 4C represents the structural alignment.

**Fig. 5:**
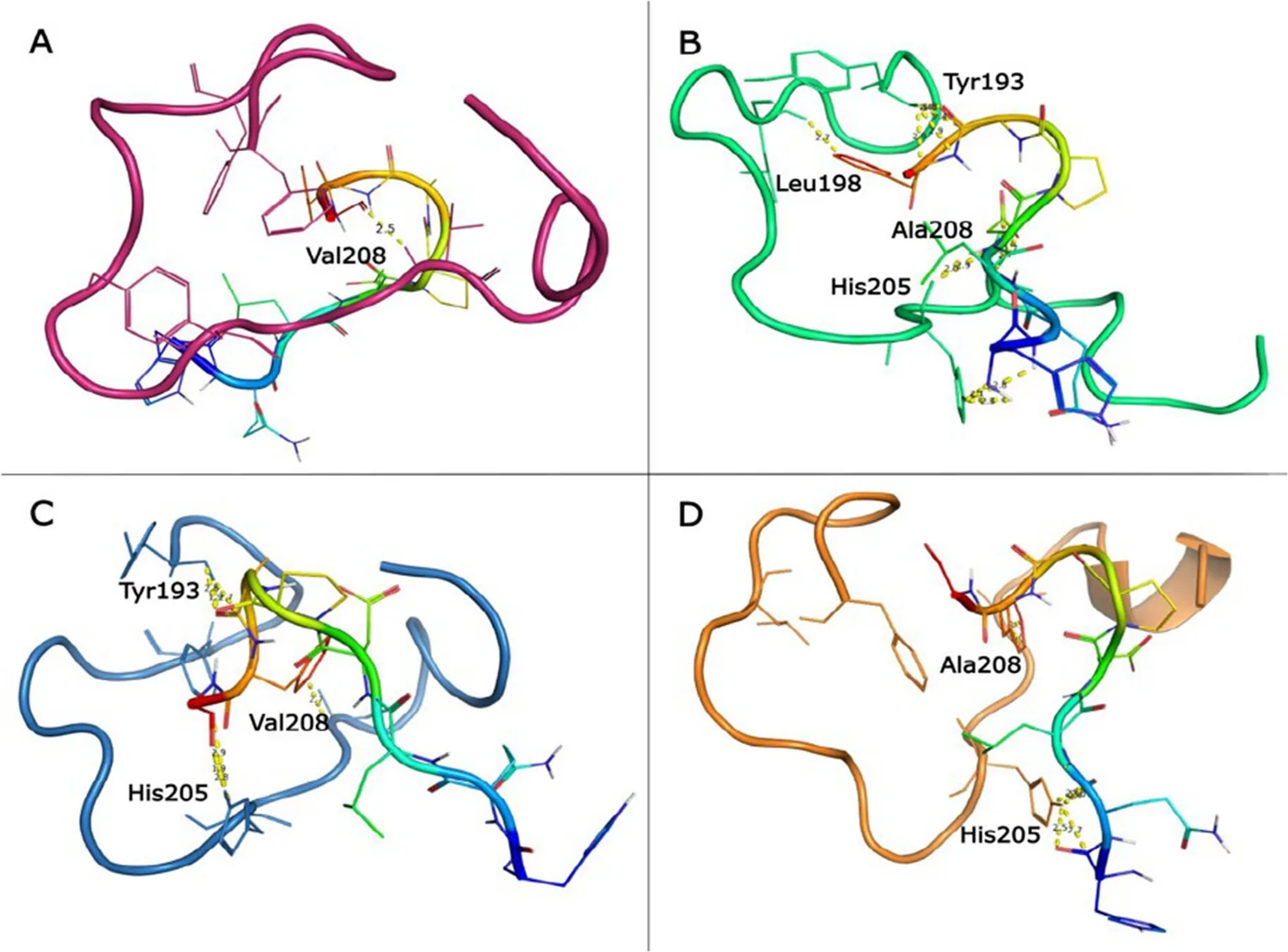
Molecular docking representations of the interaction between the C-terminal region of Large HDAg and the HBsAg peptide. (**A**) Reference structure, (**B**) Mutant1C, (**C**) MutantC2, and (**D**) Mutant3C. The ribbon cartoon represents the HBsAg peptide, while the colored structures correspond to different HDAg variants. Yellow dashed lines indicate ionic interactions between HBsAg and the C-terminal region of Large HDAg.

The docking scores of reference structure, Mutant1C, Mutant2C and Mutant3C were −62.4 ± 6.7, −70.6 ± 8.5, −78.3 ± 5.0 and −64.9 ± 1.1, respectively. The graphical representation of these interactions is shown in Figure 5. This figure highlights binding differences between reference and mutated C-terminal Large HDAg peptides when interacting with the HBsAg pre-S1 region. The structural overlays in Figure 5 indicate how specific mutations influence the positioning and binding conformation of Large HDAg, with Mutant2C exhibiting the most stable interaction, as suggested by its docking score.

## Discussion

Due to the multifactorial aspects in the pathogenesis of HDV, the investigation of genetic diversity in comparison with the different courses of infection has become relevant, which represents an important step in the investigation of this influence in patients from the Brazilian Amazon.

Our study population had a significant proportion of individuals with advanced fibrosis. HDV-3, present in all individuals, is often associated with fulminant hepatitis and death [11, 25–27]. On the other hand, one study found that patients infected with HDV genotype 1 had a lower rate of disease remission and poorer outcomes (cirrhosis, hepatocellular carcinoma or mortality) compared to patients with HDV genotype II [26]. However, there are reports of other genotypes associated with severity, such as in Russia, where patients infected with HDV-2 were found to have a severe disease (IVANIUSHINA et al., 2001). Watanabe et al. (2003) [28] showed that a genetic subgroup of HDV-4 from the Miyako Islands in the Far East was responsible for a serious illness.

In the study, several mutations were observed in the HDAg of the isolates, the majority of which were in important domains of the antigen such as RBD, NLS, CCD and VAS - virus assembly signal, which are essential for viral replication. The most frequent mutations observed in the hepatitis D virus (HDV) genome are due to the comparison with the Venezuelan reference sequence (NC_001653), isolated in 1990. This sequence represents an ancestral strain and, since then, the HDV has been subjected to selective pressures that can favor the emergence and establishment of mutations that could be considered specific genetic signatures.

Post-translational modifications (PTMs) of viral antigens, on the other hand, are gradually being seen as critical for the biological roles of viral proteins and for the modulation of positive factors by viral proteins [29]. With these observations, several lines of evidence have revealed that MPTs have pronounced effects on HDAg dynamics in terms of viral replication and virion assembly/release. According to studies on HDV-1, HDAg MPTs include methylation, phosphorylation, acetylation, sumoylation and isoprenylation (Chang et al., [30]; Li; Stallcup; Lai, [33]; Mu et al. [31, 32].

We observed the mutation Q123P/R in the phosphorylation site in the samples of the patients in the study. Although it was not the most frequent among the others, proportionally, it was a slightly frequent substitution in cases of advanced fibrosis compared to cases of mild fibrosis. Studies indicate that modifications in this position do not have a direct role in the trans-suppression activity of HDAg-L, in the assembly of empty virus-like HDAg particles and in the nuclear transport of HDAgs [31, 34]. However, there are no studies that directly link this mutation to fibrosis levels and there are no information that specifically describe the Q123P/R mutation in other HDV genotypes. Mutation S/L8P/Q/M was significant among patients with advanced fibrosis, and mutations D46E and A70T were significant among patients with normal AST levels. However, there are no studies associating these substitutions with clinical or laboratory outcomes, reinforcing the need for further research. Given the sample size (n= 35), the statistical power to associations is inherently limited. Future analyses may help assess the robustness of this association, and larger cohorts will be essential to clarify this point.

A rate of 15.1% of the F194Y substitution in HDV-3 was observed, previously reported by Kay and collaborators (2014) at a rate of 29%. This mutant strain in HDV-3 (F194Y) was related to non-F HBV genotypes, suggesting that the mutation could facilitate the association of HDV-3 with non-F HBV [35]. Nevertheless, the HBV in the study samples was not genotypically characterized to assess this association. Thus, the difference in the proportion of this mutation does not appear to have a causal relationship between the patients in the study by Kay et al. and the present study, suggesting only a genetic signature between the HDV of the study populations.

Some modifications have been in probable HDV epitopes for B lymphocytes (amino acids 2 to 17, 26 to 41, 50 to 65, 66 to 81, 106 to 121, 156 to 184 and 197 to 211) [36]. Cicero et al. (2016) [37] identified a positive selection in probable HDV epitopes in B lymphocytes that may indicate that the virus is changing to escape the host's humoral response. Delfino et al. (2012) linked mutations in amino acids located in HDAg-L (amino acids 196, 197, 198, 200, 201 and 202) to two cases of occult HBV co-infection, where these patients had positive HDV RNA for genotype 1, but non-reactive HBsAg and anti-HDV [38]. However, this profile was not found in our population.

Both the N-terminal and C-terminal regions of Large-HDAg were analyzed due to their role in HDV replication and interaction with HBsAg. Meanwhile, the interaction between the C-terminal domain of Large HDAg and the pre-S1 region of HBsAg is critical for HDV assembly and infectivity [39, 40]. The pre-S1 region drives viral entry and particle formation, acting as a key determinant for HDAg recruitment to the viral envelope [41, 42]. This interaction is mediated by the C-terminal isoprenylation motif (CXXX box), essential for HDAg incorporation into budding virions [43]. Docking analyses focused on residues 17–24 of the pre-S1 region to assess the impact of Large HDAg mutations on viral assembly.

Molecular modeling indicated that mutations in this region induced more significant structural changes than in the N-terminal, justifying the observed docking variations. This observation is particularly relevant given that the N-terminal of HDAg is known to interact with the autolytic domain of the HDV genomic RNA and attenuate its self-cleavage activity [44, 45]. Therefore, while the N-terminal region plays a structurally conserved role in mediating RNA binding, the more substantial structural deviations observed in the mutated region possibly suggest a differential functional contribution [46].

Analysis confirmed that mutated C-terminal peptides retained HBsAg binding, with some mutations enhancing affinity. This finding aligns with previous evidence demonstrating that the final 19 C-terminal amino acids of LHDAg constitute the essential packaging signal required for interaction with HBsAg. The preservation—and occasional improvement—of binding despite specific mutations suggests that the core structural determinants of the packaging motif remain functionally intact [39, 40]. Notably, MutantC2, lacking the V208A mutation, showed the highest predicted binding affinity in silico, suggesting V208A may reduce the strength of the HDAg–HBsAg interaction. Docking results reinforce this, as MutantC2 (without V208A) had the highest binding affinity, whereas MutantC1 and MutantC3, which included V208A, showed lower scores. While these in silico correlations suggest that V208A could influence viral assembly or infectivity, such functional impacts remain hypothetical. To our knowledge, no prior structural studies have specifically evaluated the effects of V208A or related point mutations in this region. Therefore, our modeling provides an initial structural framework to guide interpretation of these domains, but experimental validation will be essential to confirm these predicted effects in future work.

As this is a cross-sectional study, it is not possible to establish causal relationships between the mutations identified and possible clinical outcomes. Bioinformatics analyses predict conformational changes and molecular interactions but require experimental validation. Functional assays such as co-immunoprecipitation, microscopy, and viral replication studies will be essential to confirm these effects. Moreover, we propose that future studies incorporating site-directed mutagenesis and suitable in vitro or in vivo infection models will be essential to confirm the biological relevance of the observed variants. Overall, our findings provide a solid foundation for future investigations into HDV pathogenesis. A multifactorial approach would be necessary for a more robust association and generalization of the findings.

## Supplementary Information

Below is the link to the electronic supplementary material.

Supplementary file1 (XLSX 12 KB)

## Data Availability

All the HDV genomes analyzed in this study were deposited at GenBank under accession numbers PQ220458- PQ220492. Additional patient data are available in Supplementary File 1.
